# Undifferentiated Pancreatic Carcinoma With Osteoclast-Like Giant Cells: What Do We Know So Far?

**DOI:** 10.3389/fonc.2021.630086

**Published:** 2021-03-05

**Authors:** Pieter Demetter, Raphaël Maréchal, Francesco Puleo, Myriam Delhaye, Sébastien Debroux, Fadi Charara, Maria Gomez Galdon, Jean-Luc Van Laethem, Laurine Verset

**Affiliations:** ^1^ Department of Pathology, Institut Jules Bordet, Université Libre de Bruxelles, Brussels, Belgium; ^2^ Department of Gastroenterology, CHU Tivoli, La Louvière, Belgium; ^3^ Department of Gastroenterology, Hôpital Delta, Brussels, Belgium; ^4^ Department of Gastroenterology and Digestive Oncology, Erasme University Hospital, Université Libre de Bruxelles, Brussels, Belgium; ^5^ Department of Surgery, CHU Tivoli, La Louvière, Belgium

**Keywords:** undifferentiated (anaplastic) carcinoma, pancreas, osteoclast-like giant cells, pancreatic ductal adenocarcinoma, adenocarcinoma

## Abstract

Undifferentiated carcinoma of the pancreas is an aggressive but rare tumor for which several other terms have been used to describe its histological appearance. In addition, as osteoclast-like giant cells may accompany undifferentiated carcinoma of the pancreas, the WHO Classification distinguishes undifferentiated carcinoma with osteoclast-like giant cells (UC-OGC) from plain undifferentiated carcinoma since there are a few histopathological and clinical differences. UC-OGC was initially thought to be associated with worse prognosis compared to invasive ductal pancreatic adenocarcinoma, since it is often unresectable at diagnosis and tends to recur rapidly even if completely resected. When true UC-OGGs are carefully dissected out from other anaplastic carcinomas, it becomes, however, clear that UC-OGCs do have more indolent behavior, especially the pure UC-OGCs. This mini-review summarizes the current knowledge on UC-OGC.

## Introduction

Undifferentiated carcinoma of the pancreas is an aggressive but rare tumor for which several other terms have been used to describe its histological appearance: anaplastic carcinoma, pleomorphic carcinoma, pleomorphic large cell carcinoma, pleomorphic giant cell carcinoma, spindle cell carcinoma, sarcomatoid carcinoma and carcinosarcoma. In the current WHO Classification, all these terms are lumped together into one single category designated as undifferentiated carcinoma of the pancreas despite their histological differences (1). In addition, as osteoclast-like giant cells may accompany undifferentiated carcinoma of the pancreas, the WHO Classification distinguishes undifferentiated carcinoma with osteoclast-like giant cells (UC-OGCs) from plain undifferentiated carcinoma since there are a few histopathological and clinical differences.

Undifferentiated carcinoma of the pancreas is a rare tumor (2) and UC-OGC is a very rare tumor accounting for less than 1% of all pancreatic malignancies (3, 4). Sommers and Meissner published a first description of this tumor in 1954 (5) as an “unusual carcinoma of the pancreas”; in 1968, Juan Rosai published two cases and notified that they simulated giant cell tumors of bone (6). UC-OGC was initially thought to show worse prognosis than that of invasive ductal adenocarcinoma of the pancreas (7–9), because it is frequently found to be unresectable at diagnosis due to advanced stages (10) and tends to recur early even after complete surgical resection (11, 12). Correspondingly, median or average survival of patients with UC-OGC has been reported less than 1 year with few exceptions (9, 13–15). Another series reveals, however, a significantly better prognosis (5-year survival >50%) than conventional ductal adenocarcinoma (16). When true UC-OGGs are carefully dissected out from other anaplastic carcinomas, it becomes indeed clear that UC-OGCs do have more indolent behavior (4, 17, 18), especially the pure UC-OGCs (19).

Literature on UC-OGC is relatively scarce and largely based on case reports. In this review we mainly focus on the histological and molecular aspects of UC-OGC.

## Histopathological Features

Tumors with osteoclast-like giant cells have been reported within a variety of organs including the skin (20), breast (21), thyroid gland (22), heart (23), lung (24), and uterus (25). Within the pancreas they are usually greater than conventional pancreatic ductal adenocarcinoma with a size reaching more than 5 cm in 80% of UC-OGC at the time of diagnosis and with more than 10 cm in 50% of UC-OGC (14). UC-OGC can present an intraductal growth leading to the formation of a polypoid mass in the periampullary area, causing occlusion of the orifice of the common bile duct with jaundice and jaundice-associated symptoms (4, 16).

**Figure 1:**
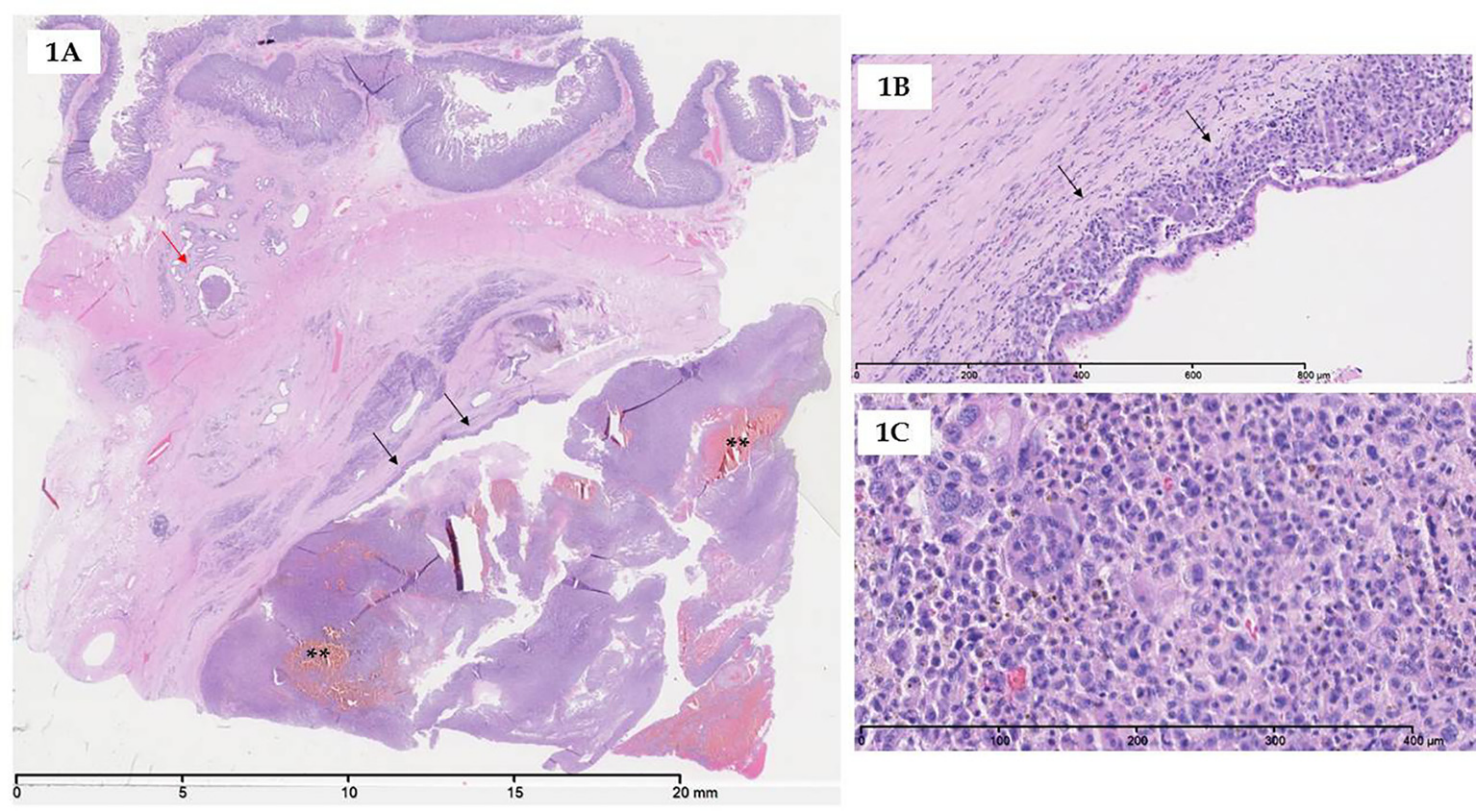
Microscopy of UC-OGC. **(A)** At low magnification, UC-OGC presenting an intraductal growth (black arrows) with few hemorragic foci (asterisks); intraductal part extending to small duct distant to the main lesion (red arrow). **(B)** UC-OGC can also present a periductal growth (black arrows); overlying epithelium exhibiting high-grade dysplasia. **(C)** UC-OGC is composed of non-neoplastic multinucleated osteoclastic-like giant cells admixed with neoplastic pleiomorphic mononuclear cells.

According to the 5^th^ edition of WHO classification, UC-OGC contains three cell types: osteoclast-like multinucleated giant cells that are non-neoplastic, mononuclear histiocytes, and neoplastic mononuclear cells (1). The former usually contain >20 uniform and small nuclei, and are often found in areas adjacent to hemorrhage or necrosis (1). Osteochondroid differentiation, osteoid and bone formation can be observed (19, 26, 27). Immunohistochemically, most of the neoplastic mononuclear cells express vimentin, some express keratin, and some label with antibodies to p53. On the other hand, osteoclast-like giant cells and a subset of the mononuclear histiocytic cells, express CD68, vimentin and leukocyte common antigen, but are negative for keratin and do not label with antibodies to p53 (8, 27–29) (**Figure 1**). The mononuclear histiocytic cells strongly and diffusely express the tumor-associated macrophages (TAM)2 marker CD163 (30).

UC-OGC can be pure or associated with another pancreatic neoplasm like intraductal papillary mucinous neoplasm, pancreatic mucinous cystic neoplasm, adenosquamous carcinoma, cystadenocarcinoma, and conventional ductal adenocarcinoma (12, 31–38).

## Molecular Aspects

Using whole exome sequencing of eight UC-OGCs, a recent study demonstrated that genetic alterations observed in UC-OGC are closely similar to those identified in carcinogenesis of pancreatic ductal adenocarcinoma and include activating mutations in the oncogene *KRAS* and inactivating mutations in the tumor suppressor genes *CDKN2A*, *TP53* and *SMAD4* (19). This finding supports current WHO classification as variant of pancreatic ductal adenocarcinoma (1, 28). The study also revealed mutations in *SERPINA3* in two cases; the presence of non-synonymous missense mutaions at the same amino residue suggests an oncogene (19). *SERPINA3* encodes alpha 1-antichimotrypsin, the most abundant component of a family of serine protease inhibitors (also known as serpins) (39). Interestingly, high SERPINA3 expression is strongly associated with worse overall and disease specific survival at 5 years in melanoma patients (40). Moreover, the protein promotes endometrial cancer cell growth (41) and SERPINA3 expression shows a rising trend in low, intermediate, and high metastatic potential colon cancer cells (42).

The detection of *GLI3* mutations in two cases suggests that *GLI3* is also a driver of UC-OGC (19). The transcription factor GLI3 is a member of the Hedgehog (Hh/HH) signaling pathway and regulates various biological processes that are important for cancer cell growth and progression (43).

Mutations in *TTN*, *MAGEB4*, and *MEGF8* have also been detected. Since these mutations all are non-synonimous missense mutations that are not clustered in any specific hotspot, the functional importance of them is, however, difficult to interpret (19).

The multinucleated histiocytic giant cells are considered as non-neoplastic because microdissected histiocytic giant cells positive for CD68 didn’t harbor *KRAS* mutations while highly neoplastic pleiomorphic mononuclear cells negative for CD68 did, bearing the hypothesis of common ductal lineage (44). However, some authors detected *KRAS* mutations in histiocytic giant cells suggesting the ability of these cells to phagocytize tumoral cells (29).

With regard to carcinogenesis of pancreatic ductal adenocarcinoma, well-known molecular alterations occur like telomere shortening, activating mutations in *KRAS*, inactivating mutations or epigenetic silencing of *p16*/*CDKN2A* and inactivating mutations in *TP53* and *SMAD4* leading to pancreatic intraepithelial neoplasia (PanIn) formation and, finally, to invasive ductal adenocarcinoma (45). As mentioned above, UC-OGC shares with pancreatic ductal adenocarcinoma the same genetic background and derives from ductal tumoral clones; however, molecular events determining the pleiomorphic phenotype of tumoral cells forming UC-OGC are currently unknown. Some authors suggest that such pleiomorphic phenotype similar to a mesenchymal phenotype is the result of epithelial-to-mesenchymal transition (EMT): Yonemasu et al. report a loss of E-cadherin in seven undifferentiated carcinomas (46), Sano et al. demonstrate a deregulation of the *β*-catenin pathway in anaplastic carcinoma (47) and, more recently, Naito et al. highlight that these cells are negative for E-cadherin and strongly positive for vimentin and ZEB1 (48) which is a pivotal element of the EMT process (49). A recent report described, however, that EMT activation is more frequent in undifferentiated carcinoma than in UC-OGC (50). Evidence of EMT activation was found in 50% of UC-OGC cases, and the frequency was higher in UC-OGC with an associated pancreatic ductal adenocarcinoma. The most strongly and frequently expressed marker in both tumor types was Snai2; this was also the most important in determining the observed differences between UC-OGC and plain undifferentiated carcinoma (50). EMT activation in UC-OGC seemed more frequent after neoadjuvant chemotherapy (50); another recent report described frequent expression of EMT-related markers in neoadjuvant-treated pancreatic ductal adenocarcinoma (51).

The pleiomorphic tumoral cells can produce granulocyte colony-stimulating factor (G-CSF) allowing recruitment of non-neoplastic OGCs (52), and high serum level of G-CSF was found in a patient with anaplastic carcinoma (53).

**Figure 2:**
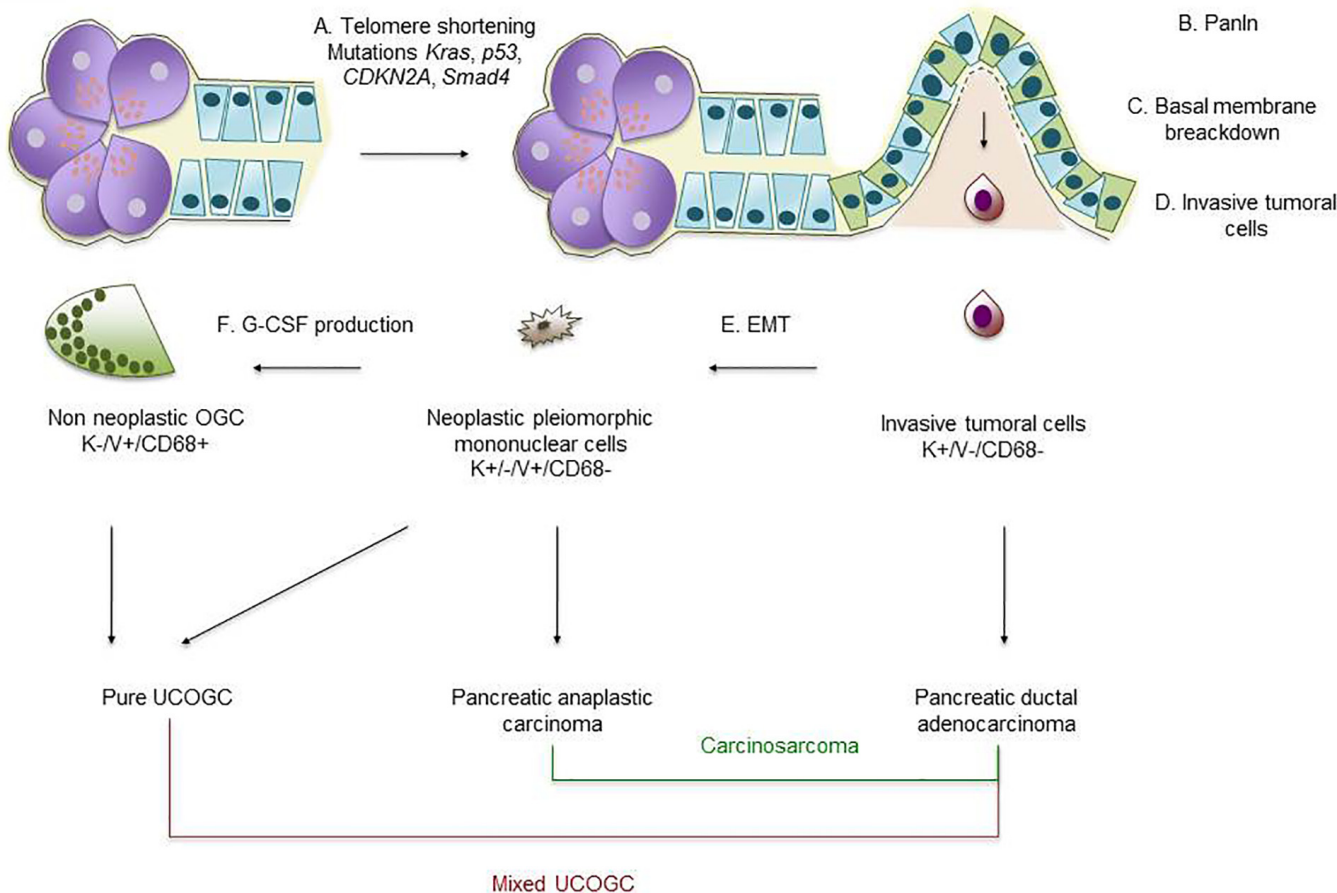
Schematic representation of pancreatic carcinogenesis. (A) Telomere shortening and *KRAS*, *p53*, *CDKN2A* and *SMAD4* mutations lead to development of preneoplastic lesions like PanIn. (B, C, D) Invasive tumoral cells of ductal origin break through the basal membrane and invade surrounding tissue; such tumoral cells (positive for keratins and negative for vimentin and CD68) will form pancreatic ductal carcinoma. (E) After EMT process (loss of E-cadherin and gain of vimentin and ZEB1), tumoral cells of ductal origin will develop a mesenchymal/pleiomorphic phenotype; such tumoral cells are associated with anaplastic carcinoma and, if a classical pancreatic ductal adenocarcinoma is present, with a carcinosarcoma. (F) Mesenchymal/pleiomorphic tumoral cells produce G-CSF recruiting multinucleated non-neoplastic OGCs; admixed pleiomorphic tumoral cells with multinucleated non-neoplastic OGCs form pure UC-OGC or, if a classical pancreatic ductal adenocarcinoma component is present, form a mixed UC-OGC. PanIn, pancreatic intraepithelial neoplasia; UC-OGC, undifferentiated carcinoma with osteoclastic-like giant cells; EMT, epithelial-to-mesenchymal transition; K, keratin; V, vimentin.

**Figure 2** summarizes the potential pathways leading to pancreatic ductal adenocarcinoma, anaplastic carcinoma, carcinosarcoma, pure UC-OGC and mixed UC-OGC.

## Diagnosis

Pancreatic ductal adenocarcinoma has been well described in terms of computed tomography (CT) and magnetic resonance imaging (MRI) characteristics. The CT and MRI characteristics of undifferentiated carcinoma of the pancreas are, however, not well known. On imaging work-up, UC-OGC appears larger than pancreatic ductal adenocarcinoma and generally displays a cystic component (14). Bile duct dilatation, pancreatic duct dilatation and necrotic areas are common findings (54, 55). Calcification (56), hemorrhage (56, 57), and venous tumor thrombus (57) have also been described. At abdominal MRI UC-OGC usually presents a low to dark signal intensity on T1- and T2-weighted images. This low-intensity appearance likely results from hemosiderin deposits in the abundant histiocytic cells of the tumor. Relatively high signal intensity in the central area may reflect the necrotic part of the tumor. Recognition that UC-OGC has a well-defined hypovascular appearance with a decreased signal on MRI may be helpful in differentiating it from other pancreatic solid lesions (54, 58). Larger studies should, however, reveal to which extent these findings are characteristic of UC-OGC.

Elevated serum levels of CEA and CA19.9 are less commonly observed than in pancreatic ductal adenocarcinoma (14). Endoscopic ultrasonography with fine-needle aspiration or biopsy provides cytological or histological material and allows immunohistochemistry (59–61). Components of UC-OGC can be identified on cytologic material. Giant cells can, however, also be detected in case of pancreatitis, representing an important differential diagnosis with UC-OGC, especially at cytology (61).

## Prognosis and Treatment

The survival of UC-OGC varies from 4 months to 10 years (27). Shiozawa et al. who summarized the prognosis of cases published until 1997, observed only two long-term survivors in a group of 32 patients (62), while Strobel et al. reported that 80% of the patients who underwent curative surgery survived for at least 2 years (63). In a more recent meta-analysis, the authors highlight that older age, male gender, small tumor, lymph node metastases, and a concomitant component of pancreatic ductal carcinoma are characteristics associated with short-term survival (64). Discordant prognosis data in the litterature is probably due to the use of wrong terminology, as mentioned before.

Luchini et al. demonstrated that the most important criterium for prognosis is the presence of an associated pancreatic ductal adenocarcinoma; they showed that median overall survival for pure UC-OGC was 36 months, compared with 15 months for UC-OGC with associated pancreatic ductal adenocarcinoma (19). This study underlines the importance of extensive sampling for histopathological examination.

Due to the rarity of UC-OGC, treatment options have never been standardized. Surgery is the first-choice treatment. The efficacity of radio- and/or chemotherapy in UC-OGC remains to be evaluated (14); since UC-OGC is considered a variant of ductal adenocarcinoma of the pancreas, standard chemotherapeutic regimens can, however, be used. FOLFIRINOX is currently preferred to gemcitabine since this results in better overall survival (65). Since patients with undifferentiated carcinoma are often in poor condition, paclitaxel-containing regimens can, however, be considered a reasonable choice; this would offer relatively long survival, as has been shown in a recent retrospective multicenter cohort study (66).

PD-1 or PD-L1 monoclonal antibody therapy has demonstrated promising therapeutic effects in clinical studies of several cancer types. This therapy has been succesful in multiple prospective randomized clinical trials on non-small cell lung cancer, renal cell carcinoma, melanoma, Hodgkin lymphoma, breast carcinoma, head and neck squamous cell carcinoma and a subset of urothelial carcinoma (67–72). It has been demonstrated that PD-L1 is expressed in neoplastic cells of about 60–80% of UC-OGC cases, and particularly in cases with an associated pancreatic ductal adenocarcinoma. Expression of PD-L1 is associated with poor prognosis (30, 73, 74). The possible mechanism underlying the aggresive behavior of PD-L1 positive UC-OGC might be the inhibition of anti-tumor immunity by PD-L1, allowing neoplastic cells to escape the cytotoxic activity of T-lymphocytes (73). This hypothesis is supported by the fact that UC-OGC contains numerous inflammatory cells (1); UC-OCG contains significantly more CD3^+^ and CD8^+^ tumor-infiltrating lymphocytes/mm^2^ than conventional pancreatic ductal adenocarcinoma (73). These insights might have potential impact for therapeutic strategies and suggest a strong need for a clinical trial of immune checkpoint immunotherapy in patients with advanced PD-L1 positive UC-OGC. Such immunotherapy may also exert antitumor effects on distant metastases of UC-OGC, as recently shown (75).

## Concluding Remarks and Future Perspectives

The identification of UC-OGC is important since this tumor has a better prognosis than conventional pancreatic ductal adenocarcinoma and than undifferentiated carcinoma without giant cells. This especially holds true for pure UC-OGC, *i.e.* UC-OGC without associated ductal adenocarcinoma. The improved survival of patients with pure UC-OGC might suggest that the unique morphology is a result of the immune response to an otherwise classical ductal pancreatic adenocarcinoma: elimination of the pancreatic ductal adenocarcinoma component by a strong immune respons could result in improved prognosis (19). Proof for this hypothesis is, however, lacking.

Recent studies have given important new insights into the clinical and molecular features of UC-OGC. Available data suggest that UC-OGC shares genetical similarities with conventional ductal adenocarcinoma, but is clinically distinct from it. Future studies should compare molecular alterations in UC-OGC and associated pancreatic ductal adenocarcinomas from the same patient to further define what is unique or typical for UC-OGC. The histopathological and clinical characteristics of this tumor type are, however, unlikely to be due to specific genetic alterations but are probably the result of gene expression or other molecular processes not related to somatic mutations (19).

Currently surgery is the first-choice treatment whereas the efficacy of radio- and/or chemotherapy remains to be evaluated. Since expression of PD-L1 is associated with poor prognosis, a clinical trial with immune checkpoint immunotherapy seems warranted. Moreover, since the neoplastic cells and osteoclast-like giant cells are surrounded by CD163^+^ TAM2 that promote survival and proliferation of neoplastic cells (30), UC-OGC may be investigated as a model for testing therapies that block or “re-educate” that macrophage population (76, 77). Whole exome sequencing might further provide potential treatment strategies for UC-OGC. Since high-quality data on this tumor type are rare, an international registry of UC-OGC would be ideal to study its pathogenesis and treatment regimen.

## Author Contributions

PD and LV contributed equally to this work, generated the figures, and wrote the manuscript. RM, FP, MD, SD, FC, MG, and JLVL contributed to the writing of the manuscript. All authors contributed to the article and approved the submitted version.

## Conflict of Interest

The authors declare that the research was conducted in the absence of any commercial or financial relationships that could be construed as a potential conflict of interest.

## References

[1] WHO Classification of Tumours Editorial Board. WHO Classification of Tumours of the Digestive System. 5th ed. Lyon: International Agency for Research on Cancer (IARC (2019) p. 328–30.

[2] Chen J Baithun SI. Morphological study of 391 cases of exocrine pancreatic tumours with special reference to the classification of exocrine pancreatic carcinoma. J Pathol (1985) 146:17–29. 10.1002/path.1711460108 2989468

[3] Jo S. Huge undifferentiated carcinoma of the pancreas with osteoclast-like giant cells. World J Gastroenterol (2014) 20:2725–30. 10.3748/wjg.v20.i10.2725 PMC3949283 24627610

[4] Maksymov V Khalifa MA Bussey A Carter B Hogan M. Undifferentiated (anaplastic) carcinoma of the pancreas with oesteoclast-like giant cells showing various degree of pancreas duct involvement: a case report and literature review. JOP (2011) 12:170–6. 21386647

[5] Sommers SC Meissner WA. Unusual carcinomas of the pancreas. AMA Arch Pathol (1954) 58:101–11. 13170907

[6] Rosai J. Carcinoma of the pancreas simulating giant cell tumor of bone. Electron-microscopic evidence of its acinar cell origin. Cancer (1968) 22:333–44. 10.1002/1097-0142(196808)22:2<333::aid-cncr2820220210>3.0.co;2-a 5660199

[7] Zou XP Yu ZL Li ZS Zhou GZ. Clinicopathological features of giant cell carcinoma of the pancreas. Hepatobiliary. Pancreat Dis Int (2004) 3:300–2. 15138131

[8] Lukas Z Dvorak K Kroupova I Valaskova I Habanec B. Immunohistochemical and genetical analysis of the osteoclastic giant cell tumor of the pancreas. Pancreas (2006) 32:325–9. 10.1097/01.mpa.0000202951.10612.fa 16628090

[9] Paal E Thompson LD Frommelt RA Przygodzki RM Heffess CS. A clinicopathologic and immunohistochemical study of 35 anaplastic carcinomas of the pancreas with a review of the literature. Ann Diagn Pathol (2001) 5:129–40. 10.1053/adpa.2001.25404 11436166

[10] Bergmann F Esposito I Michalski CW Herpel E Friess H Schirmacher P. Early undifferentiated pancreatic carcinoma with osteoclastlike giant cells: direct evidence for ductal evolution. Am J Surg Pathol (2007) 31:1919–25. 10.1097/PAS.0b013e318067bca8 18043049

[11] Wada T Itano O Oshima G Chiba N Ishikawa H Koyama Y. A male case of an undifferentiated carcinoma with osteoclast-like giant cells originating in an indeterminate mucin-producing cystic neoplasm of the pancreas. A case report and review of the literature. World J Surg Oncol (2011) 9:100. 10.1186/1477-7819-9-100 21902830 PMC3186749

[12] Hirano H Morita K Tachibana S Okimura A Fujisawa T Ouchi S. Undifferentiated carcinoma with osteoclast-like giant cells arising in a mucinous cystic neoplasm of the pancreas. Pathol Int (2008) 58:383–9. 10.1111/j.1440-1827.2008.02240.x 18477218

[13] Yoshioka M Uchinami H Watanabe G Takahashi T Nakagawa Y Andoh H. Effective use of gemcitabine in the treatment of undifferentiated carcinoma with osteoclast-like giant cells of the pancreas with portal vein tumor thrombus. Intern Med (2012) 51:2145–50. 10.2169/internalmedicine.51.7670 22892493

[14] Gao HQ Yang YM Zhuang Y Liu P. Locally advanced undifferentiated carcinoma with osteoclast-like giant cells of the pancreas. World J Gastroenterol (2015) 21:694–8. 10.3748/wjg.v21.i2.694 PMC4292306 25593500

[15] Tezuka K Yamakawa M Jingu A Ikeda Y Kimura W. An unusual case of undifferentiated carcinoma *in situ* with osteoclast-like giant cells of the pancreas. Pancreas (2006) 33:304–10. 10.1097/01.mpa.0000235303.11734.2a 17003654

[16] Muraki T Reid MD Basturk O Jang KT Bedolla G Bagci P. Undifferentiated carcinoma with osteoclastic giant cells of the pancreas : clinicopathological analysis of 38 cases highlights a more protracted clinical course than currently appreciated. Am J Surg Pathol (2016) 40:1203–16. 10.1097/PAS.0000000000000689 PMC4987218 27508975

[17] Deckard-Janatpour K Kragel S Teplitz RL Min BH Gumerlock PH Frey CF. Tumors of the pancreas with osteoclast-like and pleomorphic giant cells : an immunohistochemical and ploidy study. Arch Pathol Lab Med (1998) 122:266–72. 9823867

[18] Mannan R Khanna M Bhasin TS Misra V Singh PA. Undifferentiated carcinoma with osteoclast-like giant cell tumor of the pancreas : a discussion of rare entity in comparison with pleomorphic giant cell tumor of the pancreas. Indian J Pathol Microbiol (2010) 53:867–8. 10.4103/0377-4929.72016 21045455

[19] Luchini C Pea A Lionheart G Mafficini A Nottegar A Veronese N. Pancreatic undifferentiated carcinoma with osteoclast-like giant cells is genetically similar to, but clinically distinct from, conventional ductal adenocarcinoma. J Pathol (2017) 243:148–54. 10.1002/path.4941 PMC6664430 28722124

[20] Jimenez-Heffernan JA Adrados M Munos-Hernandez P Fernandez-Rico P Ballesteros-Garcia AI Fraga J. Cytologic features of malignant melanoma with osteoclast-like giant cells. Acta Cytol (2018) 62:151–4. 10.1159/000486027 29332062

[21] Ohashi R Yanagihara K Namimatsu S Sakatani T Takei H Naito Z. Osteoclast-like giant cells in invasive breast cancer predominantly possess M2-macrophage phenotype. Pathol Res Pract (2018) 214:253–8. 10.1016/j.prp.2017.11.002 29129494

[22] Mehdi G Ansari HA Siddiqui SA. Cytology of anaplastic giant cell carcinoma of the thyroid with osteoclast-like giant cells – a case report. Diagn Cytopathol (2007) 35:111–2. 10.1002/dc.20595 17230568

[23] Katoh M Shigematsu H. Leiomyosarcoma of the heart and its pulmonary metastasis, both with prominent osteoclast-like multinucleated giant cells expressing tartrate-resistant acid phosphatase activity. Pathol Int (1999) 49:74–8. 10.1046/j.1440-1827.1999.00817.x 10227728

[24] Dahm HH. Non-small cell carcinoma of the lung with osteoclast-like giant cells. Int J Surg Pathol (2017) 25:258–61. 10.1177/1066896916679519 PMC5405838 27899694

[25] Cheng CH Su B Ding DC. Rare case of undifferentiated uterine sarcoma with neuroectodermal differentiation and osteoclast-like giant cells. Taiwan J Obstet Gynecol (2018) 57:442–6. 10.1016/j.tjog.2018.04.020 29880181

[26] Manduch M Dexter DF Jalink DW Vanner SJ Hurlbut DJ. Undifferentiated pancreatic carcinoma with osteoclast-like giant cells: report of a case with osteochondroid differentiation. Pathol Res Pract (2009) 205:353–9. 10.1016/j.prp.2008.11.006 19147301

[27] Molberg KH Heffess C Delgado R Albores-Saavedra J. Undifferentiated carcinoma with osteoclast-like giant cells of the pancreas and periampullary region. Cancer (1998) 82:1279–87. 10.1002/(sici)1097-0142(19980401)82:7<1279::aid-cncr10>3.0.co;2-3 9529019

[28] Hoorens A Prenzel K Lemoine NR Klöppel G. Undifferentiated carcinoma of the pancreas: analysis of intermediate filament profile and Ki-ras mutations provides evidence of a ductal origin. J Pathol (1998) 185:35–60. 10.1002/(SICI)1096-9896(199805)185:1<53::AID-PATH45>3.0.CO;2-F 9713360

[29] Westra WH Sturm P Drillenburg P Choti MA Klimstra DS Albores-Saavedra J. K-ras oncogene mutations in osteoclast-like giant cell tumors of the pancreas and liver : genetic evidence to support origin from the duct epithelium. Am J Surg Pathol (1998) 22:1247–54. 10.1097/00000478-199810000-00010 9777987

[30] Luchini C Cros J Pea A Pilati C Veronese N Rusev B. PD-1, PD-L1 and CD163 in pancreatic undifferentiated carcinoma with osteoclast-like giant cells: expression patterns and clinical implications. Hum Pathol (2018) 81:157–65. 10.1016/j.humpath.2018.07.006 30031096

[31] Jang KT Park SM Basturk O Bagci P Bandyopadhyay S Stelow EB. Clinicopathologic characteristics of 29 invasive carcinomas arising in 178 pancreatic mucinous cystic neoplasms with ovarian-type stroma: implications for management and prognosis. Am J Surg Pathol (2015) 39:179–87. 10.1097/PAS.0000000000000357 PMC4460193 25517958

[32] Alwaheeb S Chetty R. Adenosquamous carcinoma of the pancreas with an acantholytic pattern together with osteoclast-like and pleomorphic giant cells. J Clin Pathol (2005) 58:987–90. 10.1136/jcp.2004.025221 PMC1770836 16126885

[33] Sedivy R Kalipciyan M Mazal PR Wolf B Wrba F Karner-Hanusch J. Osteoclast-like giant cell tumor in mucinous cystadenocarcinoma of the pancreas: an immunohistochemical and molecular analysis. Cancer Detect Prev (2005) 29:8–14. 10.1016/j.cdp.2004.10.006 15734212

[34] Watanabe M Miura H Inoue H Uzuki M Noda Y Fujita N. Mixed osteoclastic/pleomorphic-type giant cell tumor of the pancreas with ductal adenocarcinoma: histochemical and immunohistochemical study with review of the literature. Pancreas (1997) 15:201–8. 10.1097/00006676-199708000-00013 9260206

[35] Posen JA. Giant cell tumor of the pancreas of the osteclastic type associated with a mucous secreting cystadenocarcinoma. Hum Pathol (1981) 12:944–7. 10.1016/s0046-8177(81)80203-1 7298052

[36] Lane RB Jr Sangueza OP. Anaplastic carcinoma occuring in association with a mucinous cystic neoplasm of the pancreas. Arch Pathol Lab Med (1997) 121:533–5. 9167613

[37] Fujii K Nitta T Kawasaki H Kataoka J Tominaga T Inoue Y. Anaplastic carcinoma of the pancreas arising in an intraductal papillary mucinous neoplasm: a case report. Mol Clin Oncol (2016) 4:39–42. 10.3892/mco.2015.671 26870354 PMC4727070

[38] Chiarelli M Guttadauro A Gerosa M Marando A Gabrielli F De Simone M. An indeterminate mucin-producing cystic neoplasm containing an undifferentiated carcinoma with osteoclast-like giant cells: a case report of a rare association of pancreatic tumors. BMC Gastroenterol (2015) 15:161. 10.1186/s12876-015-0391-2 26581412 PMC4652416

[39] Byth BC Billingsley GD Cox DW. Physical and genetic mapping of the serpin gene cluster at 14q32.1: allelic association and a unique haplotype associated with alpha 1-antitrypsin deficiency. Am J Hum Genet (1994) 55:126–33. PMC1918218 7912884

[40] Zhou J Cheng Y Tang L Martinka M Kalia S. Up-regulation of SERPINA3 correlates with high mortality of melanoma patients and increased migration and invasion of cancer cells. Oncotarget (2017) 8:18712–25. 10.18632/oncotarget.9409 PMC5386641 27213583

[41] Yang GD Yang XM Lu H Ren Y Ma MZ Zhu LY. SERPINA3 promotes endometrial cancer cell growth by regulating G2/M cell cycle checkpoint and apoptosis. Int J Clin Exp Pathol (2014) 7:1348–58. PMC4014215 24817931

[42] Cao LC Pei XF Qiao X Yu J Ye H Xi CL. SERPINA3 Silencing Inhibits the Migration, Invasion, and Liver Metastasis of Colon Cancer Cells. Dig Dis Sci (2018) 63:2309–19. 10.1007/s10620-018-5137-x 29855767

[43] Matissek SJ Elsawa SF. GLI3: a mediator of genetic diseases, development and cancer. Cell Commun Signal (2020) 18:54. 10.1186/s12964-020-00540-x 32245491 PMC7119169

[44] Sakai Y Kupelioglu AA Yanagisawa A Yamaguchi K Hidaka E Matsuya S. Origin of giant cells in osteoclast-like giant cell tumors of the pancreas. Hum Pathol (2000) 31:1223–9. 10.1053/hupa.2000.18491 11070115

[45] Iacobuzio-Donahue CA. Genetic evolution of pancreatic cancer: lessons learnt from the pancreatic cancer genome sequencing project. Gut (2012) 61:1085–94. 10.1136/gut.2010.236026 PMC3356493 21749982

[46] Yonemasu H Takashima M Nishiyama KI Ueki T Yao T Tanaka M. Phenotypical characteristics of undifferentiated carcinoma of the pancreas: a comparison with pancreatic ductal adenocarcinoma and relevance of E-cadherin, alpha catenin and beta catenin expression. Oncol Rep (2001) 8:745–52. 10.3892/or.8.4.745 11410776

[47] Sano M Homma T Hayashi E Noda H Amano Y Tsujimura R. Clinicopathological characteristics of anaplastic carcinoma of the pancreas with rhabdoid features. Virchows Arch (2014) 465:531–8. 10.1007/s00428-014-1631-5 25031015

[48] Naito Y Kawahara A Taira T Takase Y Murata K Ishida Y. Cytopathological and immunocytochemical findings of pancreatic anaplastic carcinoma with ZEB1 expression by means of touch imprint cytology. Diagn Cytopathol (2018) 46:198–203. 10.1002/dc.23823 28940869

[49] Caramel J Ligier M Puisieux A. Pleiotropic Roles for ZEB1 in Cancer. Cancer Res (2018) 78:30–5. 10.1158/0008-5472.CAN-17-2476 29254997

[50] Mattiolo P Fiadone G Paolino G Chatterjee D Bernasconi R Piccoli P. Epithelial-mesenchymal transition in undifferentiated carcinoma of the pancreas with and without osteoclast-like giant cells. Virchows Arch (2020). 10.1007/s00428-020-02889-3 PMC7969490 32661742

[51] Wang M Estrella JS Katz MH Kim M Rashid A Lee JE. Expression of epithelial-mesenchymal transition markers in treated pancreatic ductal adenocarcinoma. Pancreas (2019) 48:1367–72. 10.1097/MPA.0000000000001432 PMC6944196 31688603

[52] Kubota N Naito Y Kawahara A Taira T Yamaguchi T Yoshida T. Granulocyte colony-stimulating factor producing pancreatic anaplastic carcinoma in ascitic fluid at initial diagnosis: A case report. Diagn Cytopathol (2017) 45:463–7. 10.1002/dc.23682 28185423

[53] Vinzens S Zindel J Zweifel M Rau T Gloor B Wochner A. Granulocyte Colony-stimulating Factor Producing Anaplastic Carcinoma of the Pancreas: Case Report and Review of the Literature. Anticancer Res (2017) 37:223–8. 10.21873/anticanres.11310 28011495

[54] Fukukara Y Kumagae Y Hirahara M Hakamada H Nagano H Nakajo M. CT and MRI features of undifferentiated carcinomas with osteoclast-like giant cells of the pancreas: a case series. Abdom Radiol (NY) (2019) 44:1246–55. 10.1007/s00261-019-01958-9 30815714

[55] Shindoh N Ozaki Y Kyogoku S Nakanishi A Sumi Y Katayama H. Osteoclast-type giant cell tumor of the pancreas: helical CT scans. AJR Am J Roentgenol (1998) 170:653–4. 10.2214/ajr.170.3.9490947 9490947

[56] Ichikawa T Federle MP Ohba S Ohtomo K Sugiyama A Fujimoto H. Atypical exocrine and endocrine panccreatic tumors (anaplastic, small cell, and giant cell types): CT and pathologic features in 14 patients. Abdom Imaging (2000) 25:409–19. 10.1007/s002610000058 10926196

[57] Togawa Y Tonouchi A Chiku T Sano W Doki T Yano K. A case report of undifferentiated carcinoma with osteoclast-like giant cells of the pancreas and literature review. Clin J Gastroenterol (2010) 3:195–203. 10.1007/s12328-010-0160-2 26190247

[58] Yang KY Choi JI Choi MH Park MY Rha SE Byun JY. Magnetic resonance imaging findings of undifferentiated carcinoma with osteoclast-like giant cells of pancreas. Clin Imaging (2016) 40:148–51. 10.1016/j.clinimag.2015.09.013 26520702

[59] Reid MD Muraki T HooKim K Memis B Graham RP Allende D. Cytologic features and clinical implications of undifferentiated carcinoma with osteoclastic giant cells of the pancreas: An analysis of 15 cases. Cancer Cytopathol (2017) 125:563–75. 10.1002/cncy.21859 28371566

[60] Speisky D Villarroel M Vigovich F Iotti A Garcia TA Quero LB. Undifferentiated carcinoma with osteoclast-like giant cells of the pancreas diagnosed by endoscopid ultrasound guided biopsy. Ecancermedicalscience (2020) 14:1072. 10.3332/ecancer.2020.1072 32863866 PMC7434513

[61] Brosens LAA Leguit RJ Vleggaar FP Veldhuis WB van Leeuwen MS Offerhaus GJA. EUS-guided FNA cytology diagnosis of paraduodenal pancreatitis (groove pancreatitis) with numerous giant cells: conservative management allowed by cytological and radiological correlation. Cytopathology (2015) 26:122–5. 10.1111/cyt.12140 24650015

[62] Shiozawa M Imada T Ishiwa N Rino Y Hasuo K Takanashi Y. Osteoclast-like giant cell tumor of the pancreas. Int J Clin Oncol (2002) 7:376–80. 10.1007/s101470200059 12494256

[63] Strobel O Hartwig W Bergmann F Hinz U Hackert T Grenacher L. Anaplastic pancreatic cancer: Presentation, surgical management, and outcome. Surgery (2011) 149:200–8. 10.1016/j.surg.2010.04.026 20542529

[64] Kobayashi S Nakano H Ooike N Oohashi M Koizumi S Otsubo T. Long-term survivor of a resected undifferentiated pancreatic carcinoma with osteoclast-like giant cells who underwent a second curative resection: A case report and review of the literature. Oncol Lett (2014) 8:1499–504. 10.3892/ol.2014.2325 PMC4156164 25202356

[65] Conroy T Desseigne F Ychou M Bouché O Guimbaud R Bécouarn Y. Groupe Tumeurs Digestives of Unicancer; PRODIGE Intergroup. FOLFIRINOX versus gemcitabine for metastatic pancreatic cancer. N Engl J Med (2011) 364:1817–25. 10.1056/NEJMoa1011923 21561347

[66] Imaoka H Ikeda M Maehara K Umemoto K Ozaka M Kobayashi S. Clinical outcomes of chemotherapy in patients with undifferentiated carcinoma of the pancreas: a retrospective multicenter cohort study. BMC Cancer (2020) 20:946. 10.1186/s12885-020-07462-4 33004032 PMC7529509

[67] Ansell SM Lesokhin AM Borrello I Halwani A Scott EC Gutierrez M. PD-1 blockade with nivolumab in relapsed or refractory Hodgkin’s lymphoma. N Engl J Med (2015) 372:311–9. 10.1056/NEJMoa1411087 PMC4348009 25482239

[68] Hamid O Robert C Daud A Hodi FS Hwu WJ Kefford R. Five-year survival outcomes for patients with advanced melanoma treated with pembrolizumab in KEYNOTE-001. Ann Oncol (2019) 30:582–8. 10.1093/annonc/mdz011 PMC6503622 30715153

[69] Brahmer JR Tykodi SS Chow LQ Hwu WJ Topalian SL Hwu P. Safety and activity of anti-PD-L1 antibody in patients with advanced cancer. N Engl J Med (2012) 366:2455–65. 10.1056/NEJMoa1200694 PMC3563263 22658128

[70] Koshkin VS Grivas P. Emerging role of immunotherapy in advanced urothelial carcinoma. Curr Oncol Rep (2018) 20:48. 10.1007/s11912-018-0693-y 29644490

[71] Schmid P Rugo HS Adams S Schneeweiss A Barrios CH Iwata H. Atezolizumab plus nab-paclitaxel as first-line treatment for unresectable, locally advanced or metastatic triple-negative breast cancer (IMpassion130): updated efficacy results from a randomised, double-blind, placebo-controlled, phase 3 trial. Lancet Oncol (2020) 21:44–59. 10.1016/S1470-2045(19)30689-8 31786121

[72] Coledas AD Bahleda R Braiteh F Balmanoukian A Brana I Chau NG. Safety and clinical activity of atezolizumab in head and neck cancer: results from a Phase I trial. Ann Oncol (2018) 29:2247–53. 10.1093/annonc/mdy411 30219915

[73] Hrudka J Lawrie K Waldauf P Ciprova V Moravcova J Matej R. Negative prognostic impact of PD-L1 expression in tumor cells of undifferentiated (anaplastic) carcinoma with osteoclast-like giant cells of the pancreas: study of 13 cases comparing ductal pancreatic carcinoma and review of the literature. Virchows Arch (2020) 477:687–96. 10.1007/s00428-020-02830-8 32424767

[74] Lehrke HD Graham RP McWilliams RR Lam-Himlin DM Smyrk TC Jenkins S. Undifferentiated pancreatic carcinomas display enrichment for frequency and extent of PD-L1 expression by tumor cells. Am J Clin Pathol (2017) 148:441–9. 10.1093/ajcp/aqx092 29069274

[75] Obayashi M Shibasaki Y Koakutsu T Hayashi Y Shoji T Hirayama K. Pancreatic undifferentiated carcinoma with osteoclast-like giant cells curatively resected after pembrolizumab therapy for lung metastases: a case report. BMC Gastroenterol (2020) 20:220. 10.1186/s12876-020-01362-4. 10.3332/ecancer.2020.1072. 32652936 PMC7353752

[76] Chen Y Jin H Song Y Huang T Cao J Tang Q. Targeting tumor-associated macrophages: a potential treatment for solid tumors. J Cell Physiol (2020). 10.1002/jcp.30139 33200401

[77] Feng X Yu W Cao L Meng F Cong M. A novel chrysin thiazole derivative polarizes macrophages to an M1 phenotype *via* targeting TLR4. Int Immunopharmacol (2020) 88:106986. 10.1016/j.intimp.2020.106986 33182070

